# Microsatellite Analysis of Geographically Close Isolates of *Cystoisospora suis*

**DOI:** 10.3389/fvets.2019.00096

**Published:** 2019-04-02

**Authors:** Anja Joachim, Bärbel Ruttkowski, Nicola Palmieri

**Affiliations:** Institute of Parasitology, University of Veterinary Medicine Vienna, Vienna, Austria

**Keywords:** swine, coccidian, population genetics, capillary electrophoresis, short repetitive elements, *Isospora suis*

## Abstract

Microsatellites are short repetitive DNA sequences of 2–6 repeats interspersed in the genome that display a rapid mutation rate and consequently show high variation between individuals or populations. They have been used to characterize population diversity and structure and the level of variation between different isolates of a number of different organisms, including apicomplexan protozoa. Currently nothing is known about the genetic variability and population structure of *Cystoisospora suis* (Apicomplexa: Coccidia), the causative agent of piglet coccidiosis, and we made use of the recently available genome of *C. suis* (strain Wien-I) to amplify microsatellite regions (ca. 300–550 bp) and evaluate the applicability of fluorescence-labeled primers to investigate amplicon length variation at high resolution using capillary electrophoresis (CE). Two phenotypically characterized isolates (Wien-I, toltrazuril susceptible; Holl 1 toltrazuril resistant) and six field isolates from Europe were compared by conventional PCR followed by agar-gel electrophoresis, Sanger sequencing, and CE (fluorescence labeling and fragment length analysis) to evaluate the applicability of the method. Four primer pairs could be identified that amplified bands of the expected size and were labeled for CE analysis. High resolution CE for size determination of PCR amplicons proved to be a reliable and simple method. It revealed high diversity of the analyzed strains, with marked differences even between two strains from neighboring swine farms. In follow-up studies, adaptation of the PCR assay to multiplexing and amplification of small DNA quantities will provide a cost-effective tool to analyse field strains to reveal geographic diversity that could be mapped to phenotypic traits.

## Introduction

Members of the class Coccidia (subphylum Apicomplexa) comprise a large number of species parasitizing a wide range of hosts including domestic animals and humans with subsequent impact on human and animal health (1). In pigs, intestinal coccidia from the genera *Eimeria* and *Cystoisospora* have been described, among which *Cystoisospora suis* (syn. *Isospora suis*) has the most significant clinical and economic impact (2). It causes diarrhea and reduced weight gain in affected suckling piglets and, in combination with other enteropathogens, can cause considerable mortality (3–9). Transmission is direct and after ingestion of oocysts the parasites undergo rapid development in intestinal cells (mostly of the jejunum) followed by excretion of oocysts after a prepatent period of about 5 days (10, 11) Oocysts sporulate within 1–2 days in the warm and humid environment of the farrowing crate. The fast life cycle and high excretion rates in piglets favor the spread of the parasite within the farrowing unit (12). So far, nothing is known about the phenotypic and genetic diversity and phenotype and genotype distribution of *C. suis* in and between farms and geographical regions.

Control of infection and disease is usually achieved by chemotherapy using the triazinone toltrazuril (https://pubchem.ncbi.nlm.nih.gov/compound/Toltrazuril), which is highly effective in both experimental and field studies, reducing oocyst excretion, and diarrhea in treated piglets compared to untreated, infected controls (see (13) for a review on this). Toltrazuril is currently registered for this purpose in 74 countries (14) and is the only effective drug available against *C. suis* (15–17).

Recently, the first occurrence of a toltrazuril-resistant strain of *C. suis* from the Netherlands was described (13), indicating that after more than 20 years of use in piglets, the drug may be losing its efficacy and leave farmers with no alternatives for efficient and reliable control. However, no predictions can be made regarding the spread of resistance, especially the risk factors of dissemination of already fully resistant isolates between herds or farms.

Since molecular tools to address the genetic diversity of *C. suis* and geographical and temporal strain distribution have not been developed, we evaluated the usefulness of microsatellite-based DNA analysis to address these issues in *C. suis*. Microsatellites and other tandem repeats are rapidly evolving, highly divergent DNA sequences that have been used to assess diversity within species and to gain insights into their genetic population structures (18, 19). Among others, they have also been used to type two close relatives of *C. suis, Neospora caninum* (20–24), and *Toxoplasma gondii* (25–27) derived from different hosts, as well as *Cryptosporidium* from humans (28). We utilized non-coding or intergenic regions of the *C. suis* genome (29), which contain different microsatellite loci on stretches of 341–389 bp to assess differences in their length for comparison of laboratory strains and field isolates of *C. suis*. The aim was to evaluate whether this could be a suitable tool to determine genetic diversity between isolates from different, but also geographically close farms and to discriminate between phenotypically different strains of *C. suis*. We hypothesized that farm-specific isolates circulate within farms, and that under high biosafety standards of modern pig production spread to other farms is limited. For the development of suitable, cost-effective methods for strain/isolate typing we compared PCR and Sanger sequencing of amplicons with fluorescent labeling and high-resolution DNA fragment length analysis (FLA) using capillary electrophoresis CE).

## Materials and Methods

### Strains and Isolates of *C. suis*

Overall, 15 biological samples from eight strains/field isolates were used. In addition to oocysts from feces, merozoites from *in vitro* cultures were isolated and analyzed from three strains. From two strains, different passages/re isolations were investigated (Table 1).

**Table 1:** Samples investigated in this study.

**Strain/isolate (description)**	**Stage**	**No**.
**Wien-I 2014**	Oocysts	1
Laboratory strain isolated 2014 in Upper Austria, in-house passage #31 (2014); toltrazuril-susceptible	Merozoites	2
**Wien-I 2017**	Oocysts	3
Laboratory strain isolated 2014 in Upper Austria, in-house passage #41 (2017); toltrazuril-susceptible	Merozoites	4
**Austria 1**	Oocysts	5
Uncharacterized field strain isolated 2018 in Upper Austria; no previous toltrazuril treatment		
**CZ 1**	Oocysts	6
Uncharacterized field strain isolated 2018 in the Czech Republic; suspected toltrazuril resistant		
**Spain 1 2014**	Oocysts	7
Uncharacterized field strain from Spain, first isolated 2014		
**Spain 1 2016**	Oocysts	8
Uncharacterized field strain from Spain, re-isolated 2016		
**Spain 1 2017**	Oocysts	9
Uncharacterized field strain from Spain, re-isolated 2017	Merozoites	10
**Spain 2**	Oocysts	11
Uncharacterized field strain isolated 2018 in Spain; suspected toltrazuril resistant		
**Holl 1**	Oocysts	12
Laboratory strain isolated 2016 in The Netherlands, in-house passage #1 (2016); toltrazuril resistant	Merozoites	13
**Holl 2**	Oocysts	14
Uncharacterized field strain, isolated 2017 from a neighbor farm to Holl-1 from The Netherlands		
**DK 1**	Oocysts	15
Uncharacterized field strain, isolated 2010 in Denmark; suspected toltrazuril resistant		

Oocysts were purified from feces of patently infected piglets. To remove fat and debris, fecal samples containing oocysts were suspended in tap water, pelleted by centrifugation (600 × *g* for 10 min) and resuspended in 25% Percoll® (Percoll, GE Healthcare, Vienna, Austria) solution in tap water. After a second sedimentation step the Percoll® solution was removed by washing in tap water as above and the oocyst suspension was sieved (mesh size 50 μm) and subsequently set up for sporulation in 2% potassium bichromate in a Petri dish under daily aeration and water replenishment at room temperature for 1 week. Sporulated oocysts were subjected to flotation in saturated salt/sugar solution, collected from the surface after centrifugation and washed four times in tap water as above. Additionally, oocysts were treated with sodium hypochlorite (250 μl NaOCl per 5 ml oocyst suspension, incubation for 10 min at 4°C) and washed three times in PBS as above. Pellets were used immediately for DNA extraction.

For merozoite production oocysts of the respective strains were excysted and used for infection of cell cultures *in vitro* [adapted from Worliczek et al. (30)]. On days 6–8 of culture, supernatants containing developed merozoites were removed, merozoites were washed with PBS, counted and stored at −80°C before DNA preparation.

DNA was prepared from approximately 2.5 × 10^5^ oocysts or 1 × 10^6^ merozoites using a PeqGold® Tissue DNA Mini kit (Peqlab, Erlangen, Germany). Oocysts were vortexed in lysis buffer glass beads for 5 min in 1 min intervals before extraction.

### Choice of Sequences, PCR, Sequencing, and CE

Microsatellite sequences were chosen from DNA contigs of *C. suis* under the following prerequisites: non-coding or intergenic regions, tandem repeats of two, three or four bases, at least two different repeats per sequence, length of product (for Wien-I): 350–400 bp. Primer pairs (Eurofins Austria, Vienna, Austria) were constructed for eight contigs containing 27 microsatellites (Table 2); for fragment length analysis (FLA) using CE, the forward primers were fluorescence-labeled with HEX at the 5′ end.

**Table 2:** Contigs (with region sizes), microsatellites (MS, numbered for each contig), numbers of replicates (RE), repeat sizes (RS) [in bp], and included microsatellite sequences.

**MS**	**RE**	**RS**	**Microsatellite Sequence**
**contig_56 (371 bp)**			
#1	7	3	GAAGAGGAAGAAGAAGAAGAA
#2	9	3	TCTTCTTCTTCATCTTTCTTCTTCTTCTT
#3	7	3	AGAAGAAGAAGAAGAAAAAGAA
#4	29	3	AGAAGAAGAAGAAGGAGAGAGAAGAGAAGGAGAAGA AGAAGGAGAGAGAAGAGAAGAAGAAGAAGGAGAGAG AAGAGAAGAAGAAGAAG
**contig_116 (371 bp)**			
#1	5	3	ATAGAAAGATAGATAGATAGA
#2	8	4	AGATAGATTGAAGATAGATAGATAGATAGATAG
#3	27	4	AGATAGATAGATAGATTAGAGATAGATAGATAGATTAGA GATAGATAGATAGATTAGAGATAGATAGATAGATTAGAG ATAGATAGATAGATTAGAGATAGATAGATAGAT
**contig_577 (358 bp)**			
#1	5	3	GAAGAAGAAGAAGAA
#2	27	3	GAAGAAGAAGAAGAAGGAGAAGAAGAAGAAGAAGGA GAAGAAGAAGAAGAAGGAGAAGAAGAAGAAGAAGGA GAAGAAGAA
#3	5	3	GAGGAGGAGGAGGAG
**contig_911 (389 bp)**			
#1	3	6	TATCTATATCTATATCTAT
#2	15	4	TATCTATCTATATCTATCTATAATCTATCTATCTATCTTTAA TCTATCTATCTATCTATCT
#3	10	2	TATATATATATATATATATAT
**contig_1510 (373 bp)**			
#1	4	4	AGATAGATAGATAGATA
#2	3	4	TACGTACGTACGTAC
#3	29	2	AGAGAGAGAGAGAGACAGGGAGAGAGACAGAGAGAC AGAGAGAGAGACAGAGAGAGAGA
**contig_3847 (374 bp)**			
#1	29	2	ATATATATATATATATATACATATACATATATATATATATGT ATATGTATATATATAT
#2	8	2	TCTCTCTCTCTCTCTCT
#3	21	4	ATAGATAGATAGATTAAAGATAGATAGATAGATAGATAG ATAGATTAAAGATAGATAGATAGATAGATAGATAGATAG ATAGAT
#4	8	4	AGATAGATAGATTAAAGATAGATAGATAGATA
**contig_8577 (365 bp)**			
#1	5	3	TCTTCTTCTTCTTCT
#2	3	3	GAGAAGAGAAGAGAAG
#3	18	2	ATATATATATATATATATACATATATATATATATAT
**contig_9000 (341 bp)**			
#1	9	4	TACATATATACATACATACCTACATACATATATACA
#2	28	2	TATATATATATATATTTGTATATATATATATATATTTGTATATAT ATATATATATA
#3	3	4	TCTATCTATCTATCT

PCR products were amplified with an Illustra Hot Start Mix RTG (GE Healthcare, Vienna, Austria). Each reaction of 25 μl contained 250 nM of each primer and 5 μl of template DNA. Cycling conditions were 94°C 2 min (hot start), followed by 35 cycles with 94°C for 30 s, 59°C for 30 s, and 72°C for 30 sec with a final extension of 72°C for 5 min.

All amplicons were pre-checked by agarose gel electrophoresis and subjected to cycle sequencing (Microsynth, Balgach, Switzerland) or CE for FLA after normalization (Eurofins, Ebersberg, Germany) using an ABI 3130 XL sequencing machine and GeneMapper® software (Applied Biosystems, Thermo Fisher Scientific, Waltham, MA, USA). To test the reliability and reproducibility of the MS analysis, PCR runs of two different preparations from two independent DNA samples were prepared from selected strains.

### Clustering of Strains According to Microsatellite Lengths

**Figure 1:**
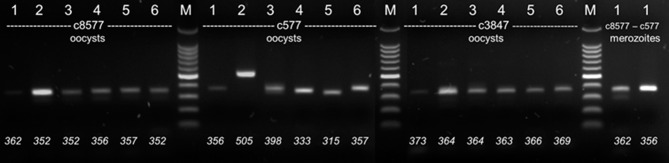
Electrophoretic separation of PCR products (agarose, 2%). DNA from six different *C. suis* strains (1: Wien-I, 2: Holl 1. 3: Spain 1, 2017 4: Holl 2, 5: DK 1, 6: Spain 1, 2014) were amplified with different primer sets (c8577, c577, c3847) from different stages (oocysts or merozoites). Bottom: Fragment sizes determined by FLA.

For each strain, a table containing the amplicon length of each locus was built (data derived from Figure 1) and loaded into R (www.r-project.org) using the function read.table (base package). A distance matrix was constructed (function dist, package stats) and the resulting matrix was employed to compute a dendrogram by hierarchical clustering (function hclust, package stats) with default parameters.

## Results

PCR products could be amplified reproducibly for four out of eight contigs, 116, 577, 3487, and 8577. The other four contigs did not yield products in the expected quality (multiple banding and/or unreliable amplification; details not shown) and were excluded from further analysis. Agarose gel electrophoresis revealed some variations between strains but not all of them could be resolved sufficiently by electrophoresis (Figure 1). Consensus sequences could only be obtained after several attempts and not for all PCR products in full length. By contrast, the exact length of the PCR product could be determined reliably by FLA using CE. Technical replicates from different DNA preparations of the same samples differed in < 2 bp in length, which is the level of accuracy stated by the manufacturer. In some isolates additional weaker bands were detected by CE (details see below). For contig 116 the amplicon was longer than predicted from the genome data, probably due to wrong assembly of the genome data (details not shown).

**Figure 2:**
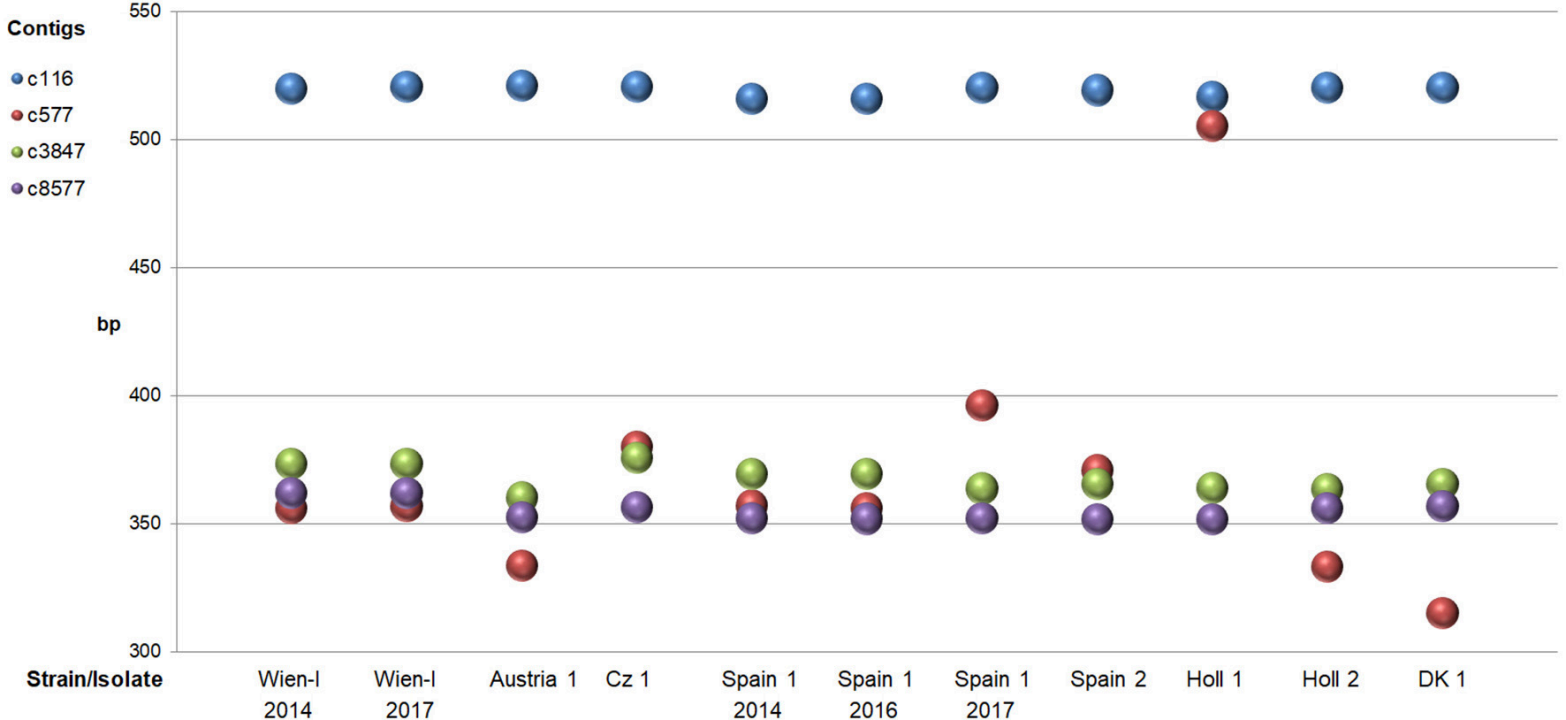
Sizes of PCR fragments (mean of duplicates) of four different microsatellite loci (contigs) amplified from eight different strains/isolates of *C. suis*. In cases where >1 fragment was present only the one with the highest amplitude in CE was taken into account.

For each of the eight different strains/isolates a unique pattern of amplicon lengths could be obtained with the four loci (Figure 2).

**Figure 3:**
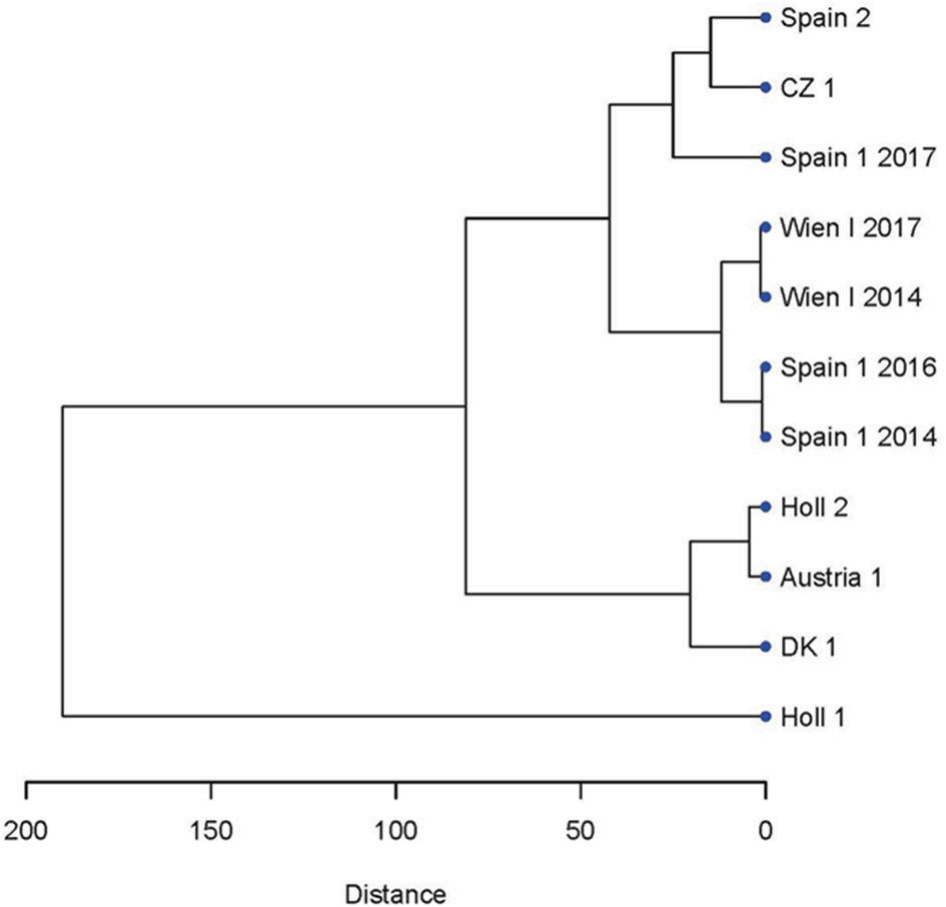
Dendrogram showing the relatedness of the strains and isolate of *C. suis* based on the microsatellite PCR fragments lengths evaluated by CE (see Figure 1).

Especially amplification of c577 produced the highest variation in length with a ~150–170 bp larger band for strain Holl 1. The difference in size was verified by sequencing showing a large insertion of about 50 replicates of the MS #2 repeats (3 bp) compared to the other strains (details not shown). Additionally, analysis of products of this primer pair by FLA showed three different bands for strain Spain 1 (Figure 3; see below, indicating that this isolate consisted of different subtypes.

To assess whether the microsatellite pattern could be related to the geography of the strains, a dendrogram was constructed by hierarchical clustering of the of the microsatellite lengths of the various strains and isolates (Figure 3). There was no apparent geographical clustering. For Spain 1 a change of pattern during the re-isolations resulted in a shift in clustering from the neighborhood of Wien-I in 2014 and 2016 to CZ 1 and Spain 2 in 2017 (Figure 3).

**Figure 4:**
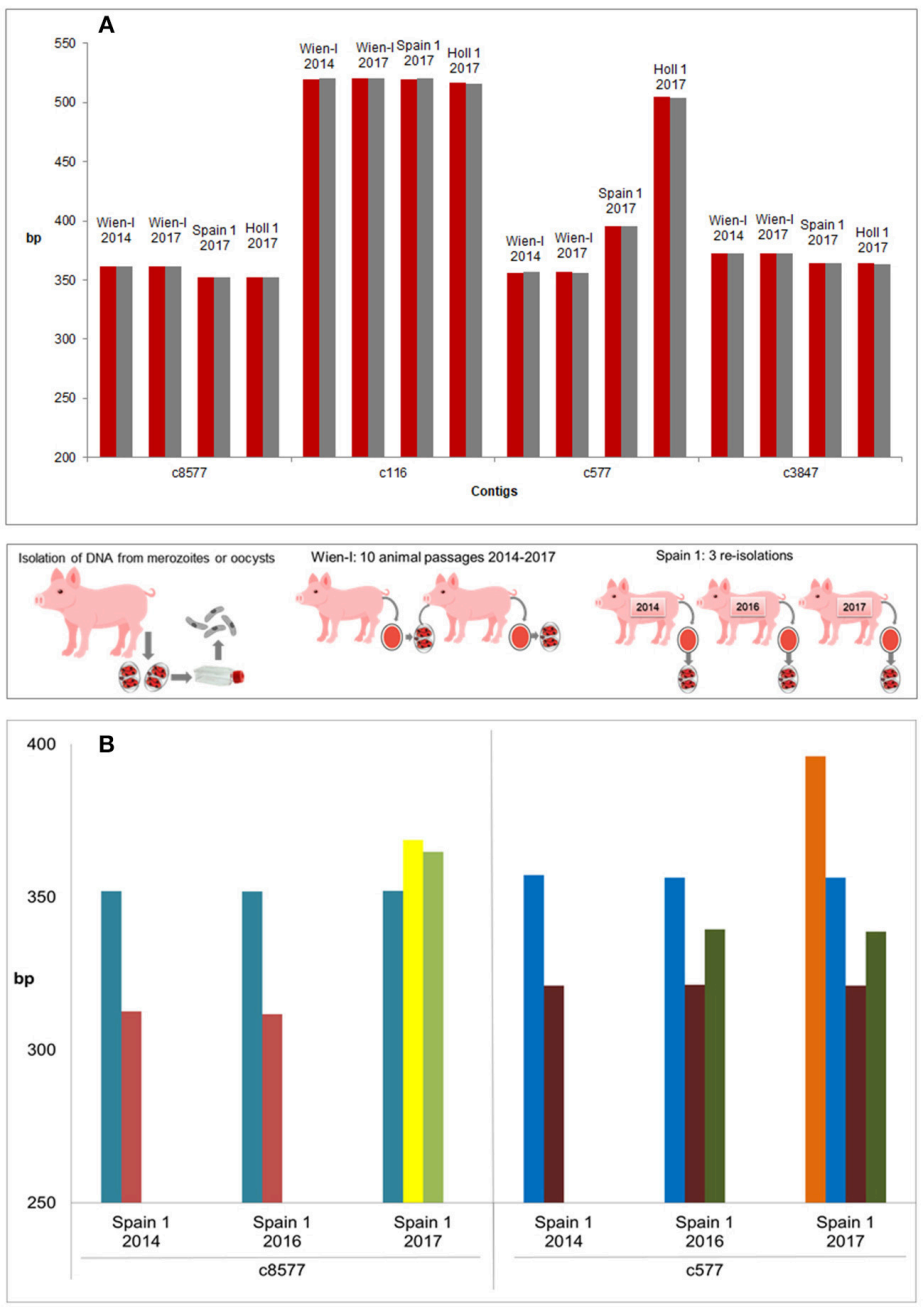
Evaluation of genetic stability and variability of strains and isolates. **(A)** Genetic stability of different stages and passages within the same strain (three strains incl. Wien I with 10 passages difference). All strains included in this experiment showed the same amplicon length for different stages (oocysts: red, merozoites: gray). No differences could be detected with the used primer pairs by FLA. **(B)** Genetic variability of three re-isolates of a field strain, Spain 1. PCR products of three independent isolations showed high variations on contigs c8577 and c577 and a clear shift of band pattern from the first (2014) to the third (2017) isolation (bands of the same length in the same color). Middle panel: Methodology.

When different stages (oocysts vs. merozoites) were compared (for samples included see Table 1) the patterns were identical between the two stages (Figure 4A).

The laboratory isolate Wien-I is continuously passaged in the laboratory of the Institute of Parasitology of the Vetmeduni Vienna since its isolation in 2005 and different passages (2014 and 2017, after 10 passages in piglets) were compared by CE to evaluate the genetic stability of the strains according to these markers. The maintained Austrian laboratory strain showed no changes in fragment lengths after 3 years of passaging (10 passages) and only one amplicon per contig (Figure 4A). By contrast, a field isolate from Spain, Spain 1, was re-isolated from the field on three consecutive time points (see Table 1 and Figure 4B) and compared. It showed mixed patterns (amplicons of different lengths per contig) and differences between the different isolation time points, indicating that Spain 1 consisted of different genotypes which shifted in the population of *C suis* on this particular farm over time (Figure 4B).

## Discussion

Microsatellites have previously been used for intra-species typing of apicomplexan parasites of the order Coccidia to determine the level of variation and for strain typing (20–27). In Apicomplexa in general, microsatellites (or single sequence repeats) are not evenly distributed within the genome and their percentage within a species genome varies greatly, which can only in part be explained by the genome sizes or gene densities (31, 32). Coccidian species with large genomes such as *T. gondii, N. caninum*, or *Eimeria falciformis* have a high percentage (>80%) of genes which contain introns and a high percentage of microsatellites (0.29–0.99% in these three species) in their genomes (31, 32), and it can be assumed that this is also the case for *C. suis* as the sister taxon to *T. gondii* and *N. caninum* (29).

For *C. suis* such analyses have not been attempted so far, although the parasite is distributed in intensive pig production worldwide and one of the leading causes of diarrhea and poor growth in suckling piglets (12). *C. suis* is a single-host intestinal protozoan which is believed to be transmitted primarily from piglet to piglet within and between litters. Older pigs, including sows, shed no or only negligible amounts of oocysts (31–35), due to extensive age resistance aided by the development of immunity after repeated infections during the suckling period (36). By contrast, suckling piglets can shed several hundred thousand oocysts per gram of feces (11, 33, 34). Under the assumption that suckling piglets are the primary source of oocysts (3, 12), the distribution of this parasite between farms is limited under high biosafety conditions when introduction of infectious agents is prevented by effective measures. This is of specific interest in cases where the parasite has developed resistance to the only available drug, toltrazuril, as recently reported (13), and the spread of the resistant parasite must be prevented and possibly also monitored.

We chose microsatellite regions from different non-coding or intergenic regions of the *C. suis* genome (29) to develop markers for strain and isolate typing. Hypervariable regions with several microsatellite sequence areas were selected from four contigs providing sequences of 300–550 bases in length. Classically, such regions are amplified by PCR and subsequently analyzed by electrophoresis (37) or Southern blot analysis (38), neither of which has a high resolution capacity. More recently melting curve analysis (39, 40) and capillary electrophoresis (41, 42) are being used as cost-effective alternatives with higher resolution capacities.

The obtained amplicons could not always be resolved unequivocally by agarose gel electrophoresis due to small size differences (in some cases only 6 bp, corresponding to two tri-nucleotide repeats), and Sanger sequencing frequently yielded only short sequences of poor quality due to the repetitive nature of the sequenced regions (43). While other methods for sequencing are more reliable, multilocus genotyping by sequencing is too labor- and cost-intensive for a large number of samples. We therefore decided to use CE in combination with FLA for suitability of amplicon characterization by size. Previous comparison of methods already demonstrated the reliability and superior high separation capacity of CE for microsatellite marker characterization (44, 45) and it has been used to type other species of coccidia (25, 45, 46). The reproducibility of the method was high with deviations of < 2 bp between technical replicates. We could assign a unique pattern of amplicon sizes to each strain and isolate used, while the used stage had no influence on the fragment sizes. The small number of microsatellite regions necessary to discern closely related strains/isolates indicates a high genetic variability of *C. suis*, similar to its sister taxa *T. gondii* (26) and *N. caninum* (20). Using contigs including several microsatellite regions increases the discriminative power (18), but with amplicon size as the only parameter, it has to be considered that gains in one and losses in another microsatellite region of the same contig can mask the differences between strains/genotypes. The presence of different alleles in the same sample was indicated by multiple banding patterns in some isolates. To fully resolve the number and composition of different alleles in a single sample further analyses such as high resolution gel electrophoresis (47) or heteroduplex tracking assays that exploit molecular probes indicating different conformational DNA structures (48) can be used.

In contrast to studies on *N. caninum* (46) geographically close isolates of *C. suis* did not cluster together, indicating that there is no regular spatial spread of genotypes; however, the number of isolates needs to be increased for valid conclusions. The long-term maintenance of a laboratory strain, Wien-I, in animals in an isolated experimental unit did not alter the pattern of amplicons, indicating that in the absence of introduction of new material, the genetic stability of the parasite within a farm is high. In some of the field isolates more than one band was amplified, indicating the presence of multiple alleles/isolates, and upon repeated re-isolation of one field isolate changes in the amplicon pattern could be observed, indicating that genetic exchange has taken place. Since the biosafety level of this farm is low, introduction of new parasite material from outside seems likely. Under isolated conditions, the parasite seems to circulate mainly within an individual farm, as was the case for the toltrazuril-resistant isolate from the Netherlands which did not spread to the neighboring farm so far (Joachim, unpublished). This would slow down the dissemination of a toltrazuril-resistant strain. Nevertheless, monitoring of the phenotype as well as the genotype is advisable to extent the control of resistance spreading in *C. suis*, especially in the light of the limited availability of alternative drugs for the control of cystoisosporosis in suckling piglets. In avian *Eimeria* species (which are phenotypically more distantly related to *C. suis* but comparable in biology and have developed considerable resistance to anticoccidial compounds over time), genotype variations have been determined for geographically different isolates of *Eimeria tenella* from China which also differed in their drug susceptibility phenotypes (49). In India, geographical segregation of avian *Eimeria* species and strains was also observed but presumed to be related to production type and farm size (50). Although the data from the present study are not sufficient for unequivocal conclusions, it can be hypothesized that the exchange of genotypes of *C. suis* between farms also differs depending on the management, especially the level of biosafety. In contrast to the studies from Asia, the genetic diversity of avian coccidia from the UK and Ireland as well as from Australia was low (51, 52). Despite the restricted possibilities to directly compare these studies with the present one due to different methodologies, genetic variability of coccidia (including avian *Eimeria* spp. and *C. suis*) has implications for the development and spread of drug resistance in these organisms and the diversity of antigens considered as vaccines (53, 54).

## Conclusion and Outlook

Currently nothing is known about the genetic variation in *C. suis*. Microsatellites provide highly variable parts of the genome that can be used to type different isolates of this parasite by FLA-CE. Although we still need to test more strains/isolates and more contigs to verify our observations, our results indicate that isolates from geographically close areas (in case of the Dutch strains, even neighboring farms) reveal high genetic diversity in the analyzed microsatellite regions. This may be due to circulation of strains within but not between farms. To determine the distribution of strains it is of relevance to estimate the risk of spreading of highly virulent or resistant isolates. In the future, this method can be developed further for higher throughput. The chosen marker sequences can be analyzed by multiplex PCR using different fluorescent labels for CE to analyse a larger set of samples with a lower yield of DNA.

## Author Contributions

AJ, BR, and NP planned the project. BR prepared the parasite material, ran the PCR and compiled the PCR and FLA data. NP did the bioinformatics analysis. AJ drafted the manuscript, BR and NP revised it. All authors agreed on the final version of the submitted manuscript.

### Conflict of Interest Statement

The authors declare that the research was conducted in the absence of any commercial or financial relationships that could be construed as a potential conflict of interest.
